# Influence of Structural Porosity and Martensite Evolution on Mechanical Characteristics of Nitinol via In-Silico Finite Element Approach

**DOI:** 10.3390/ma15155365

**Published:** 2022-08-04

**Authors:** Josiah Cherian Chekotu, David Kinahan, Russell Goodall, Dermot Brabazon

**Affiliations:** 1 Advanced Metallic Systems Centre for Doctoral Training, I-Form Advanced Manufacturing Research Centre, Dublin City University, D09 NR58 Dublin, Ireland; 2 Advanced Processing Technology Research Centre, School of Mechanical and Manufacturing Engineering, Dublin City University, D09 NR58 Dublin, Ireland; 3 Advanced Metallic Systems Centre for Doctoral Training, Department of Materials Science and Engineering, University of Sheffield, Sheffield S1 3JD, UK

**Keywords:** Nitinol, phase transformation, superelasticity, finite element analysis (FEA), martensite evolution, stiffness, porosity

## Abstract

Nitinol (NiTi) alloys are gaining extensive attention due to their excellent mechanical, superelasticity, and biocompatibility properties. It is difficult to model the complex mechanical behavior of NiTi alloys due to the solid-state diffusionless phase transformations, and the differing elasticity and plasticity presenting from these two phases. In this work, an Auricchio finite element (FE) model was used to model the mechanical behavior of superelastic NiTi and was validated with experimental data from literature. A Representative Volume Element (RVE) was used to simulate the NiTi microstructure, and a microscale study was performed to understand how the evolution of martensite phase from austenite affects the response of the material upon loading. Laser Powder Bed Fusion (L-PBF) is an effective way to build complex NiTi components. Porosity being one of the major defects in Laser Powder Bed Fusion (L-PBF) processes, the model was used to correlate the macroscale effect of porosity (1.4–83.4%) with structural stiffness, dissipated energy during phase transformations, and damping properties. The results collectively summarize the effectiveness of the Auricchio model and show that this model can aid engineers to plan NiTi processing and operational parameters, for example for heat pump, medical implant, actuator, and shock absorption applications.

## 1. Introduction

Shape memory materials possess a unique property by which they can recover a programmed shape after deformation when a mechanical or thermal force is applied. This functional property is highly utilized in engineering applications such as smart structures, sensors/actuators, energy recovery systems, biomedical, and aerospace components. Nitinol (NiTi), an intermetallic alloy of nickel and titanium in near-equiatomic compositions, exhibits the shape memory effect (SME) and superelasticity. Unlike other shape memory materials, NiTi has high ductility and mechanical strength, low stiffness, good corrosion resistance, and wear resistance. In addition to these properties, NiTi is highly resistant to pulsatile flow fatigue caused by body kinematics. It also possesses low thrombogenicity and high biocompatibility, making it ideal to be used for medical applications as stents and implants [1]. The functional properties, including shape memory effect and pseudoelasticity (superelasticity), depend primarily on the phase transformation temperatures which vary with the percentage composition of Ni and Ti. NiTi processed with higher Ti content results in martensitic (B19’ monoclinic crystal structure) phase at room temperature, which translates into higher transformation temperatures and a prominent shape memory effect. A matrix with higher Ni content results in austenitic (BCC B2 crystal structure) phase at room temperature, possesses lower transformation temperatures, and exhibits superelastic properties [2,3,4]. Martensite is characterized by needle-like crystals arrayed in a herringbone shape. The austenite phase is hard and stiff, while martensite phase is softer, more ductile, and has a lower yield stress. In some grades of NiTi, an intermediate R-phase may be present which has a rhombohedral structure exhibiting low transformation strain, and low temperature hysteresis (1–10 °C) [5,6]. The R-phase formation can be linked to any previous cold working or aging of Ni-rich alloys or may be due to alloying with an additional element like iron [3].

**Figure 1:**
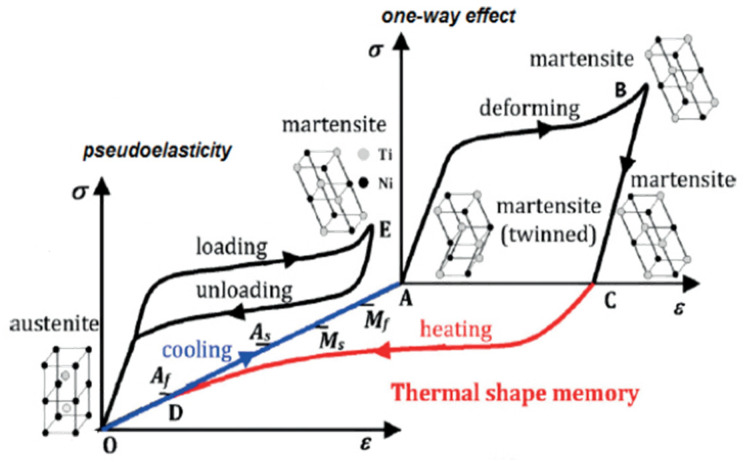
Stress–strain curve for shape memory and superelasticity of NiTi [10].

At room temperature, the shape memory NiTi will be in twinned martensite phase—see point A in Figure 1. When a deformation is applied, the phase changes to detwinned state (B) by reorienting and detwinning the lattice structure. The twin boundaries in martensite shift such that they orient in one preferential direction to better accommodate the load, referred to as “detwinning”. This microstructural process enables NiTi to withstand high strain without any permanent deformations [7]. When this detwinned martensite (C) is heated to exceed the austenite start temperature (As), austenite begins to form (D), and once the temperature crosses the austenite finish temperature (Af), the austenite formation will be complete. If an intermediate R-phase is present, cooling the cubic austenite phase results in one of the lattice diagonals elongating via a reduced angle (<90°) rhombohedral structure. If the material is cooled below the critical R-phase temperature, R-phase crystals may form. The resulting microstructure will contain both austenite and R-phase, and is referred as the pre-martensite phase [8,9]. When the material is further cooled down to the martensite start temperature (Ms), the martensite phase starts to form. The austenite (low strain phase) to martensite (high strain phase) transformation will be complete once the temperature falls below the martensite finish temperature (Mf).

These phase transformations are diffusionless shear (solid-state) transformations, which means the transition occurs through a coordinated motion of a large number of atoms relative to their neighbors. A new crystal structure is formed from parent phase without any change in the composition [3]. Superelasticity refers to the ability of the material to recover its original shape even after large deformations of 10–15% strain [11,12]. At room temperature, a superelastic NiTi will be in austenite phase. When stress is applied beyond the start of martensitic phase transformation, distortion of the crystal lattice occurs. This is associated with low hardening, and the lattice transforms completely into martensite. The Clausius–Clapeyron stress–temperature relationship for NiTi describes the activation process of forward transformation under stress from austenite to martensite, as well as the reverse transformation (martensite to austenite) [13]. This relationship indicates that the activation temperatures (As, Af, Ms, and Mf) increase linearly in the given order per unit stress [14,15,16]. At higher temperatures, martensite is unstable, and therefore returns to the austenite phase on unloading. This large elastic response of reversing the deformation to the original shape is called superelasticity. The crystalline phase of detwinned martensite occurs at lower temperatures and higher stresses, whereas the crystallization of austenite occurs at higher temperatures and lower stresses [17].

**Figure 2:**
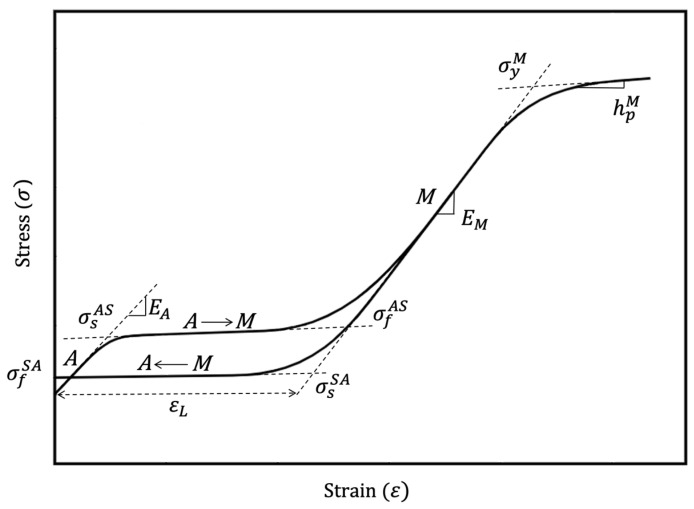
Schematic illustration showing the nomenclature used during phase transformations and mechanical loading. $E_{A}$—Young’s modulus of austenite (*A*) phase; $E_{M}$ —Young’s modulus of martensite (*M*) phase; ε_*L*_—maximum longitudinal strain; $\sigma_{s}^{AS}$ —stress to start martensitic transformation (austenite to single-variant martensite); $\sigma_{f}^{AS}$ —stress at finish of martensitic transformation; $\sigma_{s}^{SA}$ —stress to start reverse transformation (single-variant martensite to austenite); $\sigma_{f}^{SA}$ —stress at finish of reverse transformation; $\sigma_{y}^{M}$ —martensite yield stress; $h_{p}^{M}$ —martensite hardening parameter.

As seen in Figure 1 and Figure 2, when the material is deformed at constant (room) temperature, initially it follows Hooke’s law with the stiffness as that of the austenite phase. As the strain increases, a solid-state diffusionless phase transformation from austenite to martensite will occur, gradually forming a fully stress-induced martensite phase. When the strain is further increased, Hooke’s law is again followed with the stiffness of the martensite phase. After reaching the maximum elastic strain limit, detwinning/yielding occurs creating a residual strain. If the material is unloaded within the elastic strain limit, martensite phase reverts to austenite. Both forward and reverse transformations are associated with latent heat release and absorption, respectively. Full-field measurement methods, such as Infra-Red Thermography (IRT) and Digital Image Correlation (DIC), can provide quantitative information about the temperature and displacement, respectively, at each point on the surface [18,19].

The high latent heat capacity of NiTi SMAs has been found to have good potential in energy harvesting through heat transfers. These applications require complex structures to maximize the surface area for maximum energy absorption/release. These complex designs can be manufactured using the laser powder bed fusion (L-PBF) process to tailor the shape memory or superelastic effect specifically for the operating conditions [20,21,22,23,24]. The L-PBF processing of NiTi is still in the early stages of development. The response of the NiTi system to mechanical deformations can indicate the amount of work done (energy output) during the martensitic transformation. As the surface area of the structure varies, the work done will also vary, and it is vital for understanding the overall performance of the NiTi structure for renewable energy applications. It is well-addressed in literature that the L-PBF fabrication is often associated with process-induced or gas-induced porosities [25,26,27]. Besides defective porosity, application-specific porous designs can also be engineered through the L-PBF technique. For instance, in energy harvesting applications, complex designs could help to increase the surface area exposed to the fluid medium and could also help heat transfer by increasing turbulence in the fluid. In medical applications of NiTi, stiffness mismatch between the NiTi implant and surrounding bone structure reduces the long-term stability of bone implants and skeletal reconstructions. This issue could potentially be resolved by deliberately engineering porosity into the NiTi structure to lower the stiffness and match better with the bone stiffness (12–17 GPa). Some porous structures could also permit ingrowth of bone and thus better mechanical fixation [28,29].

### 1.1. Kinematics of Phase Transformations

The mechanical response of NiTi can be divided into three regimes: elastic regime, phase transformation regime, and plastic regime. In the elastic regime, the deformation causes local atomic arrangements to be continuously varied (reversible), proportional to the change in applied stress, whereas the twinning and martensitic transformation can be considered as discontinuous and diffusionless reversible changes of local arrangement of atoms propagating through the crystal as twin plane interfaces and habit planes when stress/temperature is varied. Habit planes are well-defined interfaces or contact plane between the martensite and austenite phases—these do not experience any distortion. Here, the discontinuous finite strains formed are fully recoverable under thermomechanical loads [30].

A more thorough understanding of martensitic transformation can be based on changes that happen in the crystal unit cell. During phase transformation, the atoms are moved to their new locations (shuffling). Simultaneously, space is created to accommodate the resulting structure. Since the transformation is diffusionless, time dependency can be ignored. The resulting structural change stretches the unit cell in certain directions while others are shortened. These directions do not lie on a cell edge; instead, they are irrational directions, resulting in different tension and compression levels [4]. If the transformation has no preferred direction, then the martensite opts for any of the different habit planes that exist and forms a series of crystallographically equivalent variants [31]. This results in multiple-variant martensite characterized by a twinned structure. In contrast, if there is a preferred direction, then all of the martensite crystals tend to opt for the most favorable habit plane. The product phase will then be called single-variant martensite, characterized by a detwinned structure. In addition to this, there is a possibility of conversion of each single-variant martensite into other different single-variants. This reorientation process is generally linked to non-proportional stress changes [31].

**Figure 3:**
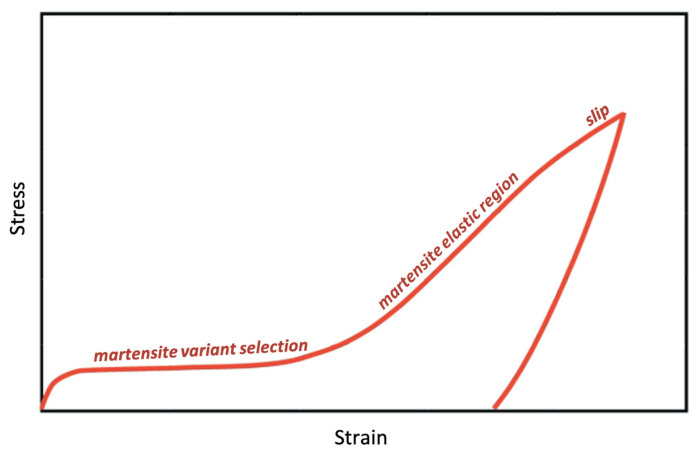
Stress–strain curve illustrating the various yield regions.

Since the austenite has a well-ordered BCC structure, only one variant will be present, while the martensite can form even 24 variants depending on the transformation history [32]. The martensite generally forms imaginary plates called correspondence variant pair (CVP) that denotes growth of two twin-related variants. The nucleation of each CVP begins with a shear stress in a direction parallel to the most favorable habit plane of each crystal. The 12 CVPs (24 variants) produce 12 distinct distortional strains, self-accommodating to the size and shape of its austenite parent phase cell [4,33]. Figure 3 shows the two distinct yield regions in a stress–strain curve of NiTi. The first yield region indicates the selection of preferred martensitic variants during phase transition, and the second yield region occurs after the detwinning event, which results in conventional slip causing plastic deformation.

It is also commonly found that stresses to initiate the phase transformation decrease as the number of cycles are increased. This is due to the internal stresses related to plastic deformation during cycling that favor martensite formation [34]. Additionally, it is seen that if the stress direction is changed, martensite reorientation occurs, resulting in a slight change in load. Transformation usually results in a volume increase; hence, less stress is required to produce the transformation in tension than in compression. This may cause asymmetry in stress–strain curves generated during tension and compression [35,36].

**Figure 4:**
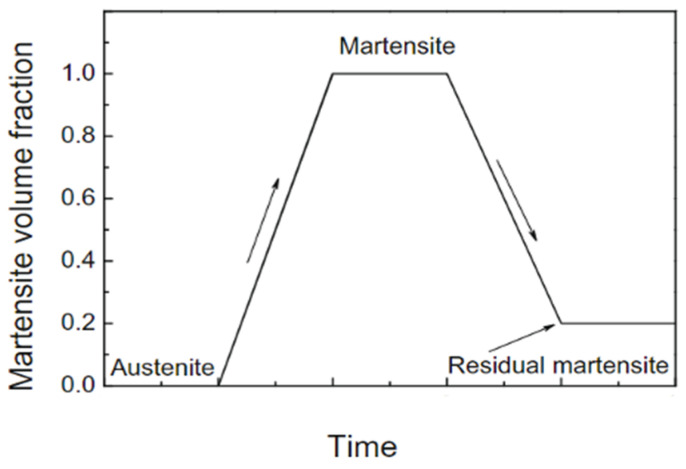
The evolution of martensite during mechanical loading and unloading in the first cycle [39].

Cyclic loading of NiTi often involves hysteresis, which is a result of the complex interactions between material grains and competing energies between grains and phases. This can degrade stiffness when the material is loaded cyclically. Strength degradation is noted when a reduction in response is observed when the same displacement level is applied. However, permanent strain addition can also happen if any residual martensite exists after each cycle causing incomplete reverse transformation. A schematic representation of the evolution of the martensite volume fraction (MVF) is shown in Figure 4. Residual martensite reduces the grain size rearranging the microstructure and hinders the motion of small grains. This ultimately results in a stronger NiTi material [37,38,39].

The martensite variant selection was also found to affect the mechanical property to a large extent. A study conducted on single crystal NiTi [40] found that tension/compression leads to an increase/decrease of Young’s modulus depending on the axis orientation of load applied and the crystal. This can be attributed to the type of variant selected under the applied force.

The complex problem in studying the change in stiffness is homogenizing the elastic properties and then estimate an effective elasticity of mixture of austenite and martensite phases (variants) in transforming NiTi. Common rule-of-mixtures approaches are Voigt and Reuss average equations [30,41,42], shown in the Appendix A section. These rules can be implemented in FEA code for estimating the change in Young’s modulus as the phase transforms from austenite to martensite.

Numerical methods, including finite element analysis (FEA) methods, allow the analysis of complex problems, comprising intricate geometries, interactions, nonlinearities, and dynamic conditions. In the past, FEA models have been used to study the response of NiTi components, such as when printing stents and actuator springs [43]. Several works have been conducted to evaluate the post-implantation structural and functional response [44,45,46], and degradation and fatigue-related studies [47,48,49].

The current work compiles a multiscale in-silico finite element (FE) model to simulate the mechanical behavior of superelastic NiTi. The associated mechanical stress–strain curves can indicate the amount of energy transfer during the phase transformations. The macroscale analysis also studies the effect of porosity on mechanical responses. Stiffness is an important mechanical property influencing the structural integrity and maximum operational strain levels in heat pump applications. These can be classified as the structural stiffness and the material stiffness (Young’s modulus). The structural stiffness is studied in the macroscale analysis, whereas a microscale analysis is performed to comprehend the material stiffness variation with respect to the phase volume fractions in the microstructure. In the case of shock absorption applications, the damping/self-centering properties of the porous structures were analyzed in terms of damping ratio and apparent stiffness calculated from the macroscale mechanical responses.

**Figure 5:**
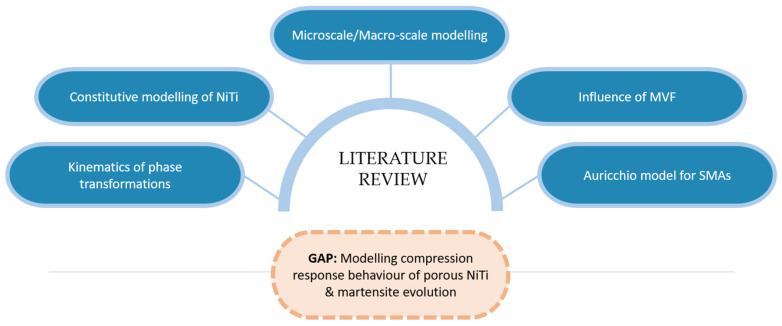
Schematic of the literature review content. Based on the gap in the literature in compressive response modelling of NiTi SMA, the scope of the current original work presented in this paper on the macroscale and microscale modelling of NiTi.

In the past, the effect of porosity and martensite volume fraction on mechanical behavior of austenitic (superelastic) NiTi has not been studied in detail from the experimental or modelling perspectives. Additionally, their influence has not been examined from an application perspective particularly for heat pumps and shock absorption applications for which SMAs have great potential. Furthermore, the effect of MVF on Young’s modulus of NiTi has not been studied for a step-by-step increment of MVF and compared with the macroscale mechanical responses. This detailed study contributes to this knowledge gap, and will help in deciding the NiTi processing conditions, model designs, and operational parameters to achieve the appropriate level of porosity for enhanced performance in shock absorption, heat pump, and other related renewable energy applications. Figure 5 summarizes the literature of this area, and different aspects of the gap examined under the scope of this current study.

## 2. Materials and Methods

### 2.1. Constitutive Modelling of Nitinol SMAs

**Figure 6:**
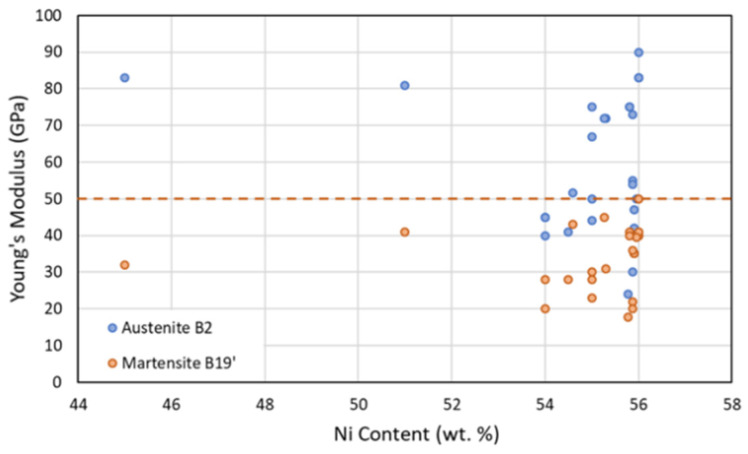
Scatter plot showing elastic modulus of the martensite and austenite phases plotted with the Ni content, from past published sources [1,34,36,38,40,50,51,52,53,54,55,56,57,58,59,60,61,62,63,64,65,66,67,68,69,70,71,72,73].

As discussed above, due to the complex material behavior in NiTi, models developed to predict the stress strain response need to take account of the major material property parameters of NiTi. The important parameters which define a NiTi material are shown in Figure 2. These parameters depend on the material compositions (Ni:Ti ratio), microstructure, mechanical and thermal histories of the material. Due to this, a wide range of mechanical and functional properties can be seen in the literature. A scatter plot of Young’s modulus of austenite and martensite phases found from various sources is shown in Figure 6. With a limited availability of sources, no specific trends were found, however, it was seen that the Young’s modulus of the martensite phase was always recorded as being below 50 GPa.

Mathematical models have provided a considerable insight into the complex mechanical behavior of NiTi. Most of the models in the literature have not been able to simulate the following prevalent NiTi phenomena: cyclic instability of superelasticity, actuation instability, shape setting, two-way SME, and functional thermomechanical fatigue [43,74]. Several constitutive models have been created over the years to capture this complex behavior, following either of the two approaches—micromechanical (microscale) or phenomenological (macroscale).

### 2.2. Microscale Models

The micromechanical model considers the granular microstructure, and describes the concepts such as nucleation, twin growth, and interface motion. The models can be used to utilize the response of single crystal based on variants for creating a polycrystalline model, by assembling single crystal grains [75]. Commonly in NiTi systems, there is a possibility of 12 product variants and 192 habit planes in each grain that constitutes the larger scale. This is often a cumbersome task from a computational perspective, and therefore, numerous constraint equations are employed for minimizing the potential issues in regard to phase fractions, mass conservation, grain boundary conditions, grain interactions, and so on [76,77,78].

Constitutive models incorporating the habit plane variants (HPVs) and CVPs as basic deformation units were used by researchers in the past [31,76,79,80,81,82,83], assuming infinitely large material. The primary focus was on evolution of dynamic microstructure during phase transformations. The first transforming grains were those with the highest Taylor and Schmid factor, and highest transformation strain. When plasticity is incorporated, it is often difficult to capture the evolution of static microstructure (dislocation defects, residual strain, austenite twins, etc.) [4,30]. Incorporating additional variables based on experimental data can help translate these micro-scale models to be used in macro-scale analysis [30,84,85,86,87,88,89,90]. Prediction models incorporating martensitic microstructure mathematical theory and crystal plasticity was suggested by recent research [30] to simulate coupled transformation-plasticity phenomenon. The work involved coupling at intrinsic lattice scale, grain-scale, and macro-scale. Boyd and Lagoudas [91] developed a constitutive model to describe the transformation and reorientation of martensite in a polycrystalline NiTi material. Huang and Brinson [92] used a multivariant model based on micromechanics and thermodynamics for single crystal NiTi. Thermomechanical modelling of simultaneous martensitic transformation, coupled with plasticity of NiTi polycrystals, is also an emerging research area.

It is often difficult to create a mathematical model to completely describe the micromechanics as there is not a sufficient extent, or easy availability, of microscale details. Additionally, a model for an engineering application often includes millions of grains, demanding large number of constraint conditions and solution variables, which then translates into heavy computational requirements. Due to these reasons, the microscale approach is not commonly used; rather a much larger phenomenological approach is preferred for engineering applications.

### 2.3. Macroscale Models

In comparison to the micromechanical approach, the macroscale constitutive models are much more pragmatic in terms of applicability and features considered. The macroscopic energy functions, which depend on internal state variables, are generally considered to obtain the response in these models. The evolution is simulated via the second law of thermodynamics. These models seek solutions to boundary value problems on the structural level through energy minimization, similar to classical plasticity models [75]. The early models were based on thermodynamics and MVF as an internal state variable to account for microstructure influence [92,93]. These formulations were based on Helmholtz free energy or Gibbs free energy.

One of the most commonly used models was created by Auricchio [31]. 1D and 3D models were created to perform superelasticity, and rate independent models. In this study, the Auricchio model [31] was implemented in the Ansys FEA platform for the shape memory analysis. The rate independent 3D model assumes a single-variant martensite, utilizes an exponential hardening law, and accounts for martensitic transformations and martensitic reorientation. The model also includes the dependency of elastic modulus on MVF. This also facilitates an insight into material strength with respect to MVF at both micro and macroscale in this study. The Auricchio model can be divided into three segments:

1. 1D and 3D constitutive model to reproduce superelasticity
2. Time-discrete isothermal model
3. Algorithmic implementation with a finite element (FE) framework.

In the constitutive model, uniaxial loading-unloading history is considered in the time-discrete model to open up the possibility of computing a closed-form solution for evaluating accuracy of FE scheme. In a real uniaxial mechanical test, an asymmetry between tension and compression response (discussed earlier) is possible. The different modes are found to exhibit different strain levels [94]. This concept is proposed using a parameter ∝, which is calculated (Equation (1)) from the initial values of phase transformation in tension and compression mode.

(1) $$\propto =\sqrt{\frac{2}{3}}\;\frac{{\left(\sigma_{S}^{AS}\right)}_{C}-{\left(\sigma_{S}^{AS}\right)}_{T}}{{\left(\sigma_{S}^{AS}\right)}_{C}+{\left(\sigma_{S}^{AS}\right)}_{T}}$$

A few important equations of the constitutive model for the shape memory property of NiTi proposed by Auricchio [31] are presented below. The most important phenomenon considered here is the phase transformation.

Internal variables considered are u, scaled transformation strain, and ξ, single-variant martensite fraction. The phase transformations can be broken down into three as:

- Conversion of austenite into single-variant martensite (A→S)
- Conversion of single-variant martensite into austenite (S→A)
- Reorientation of the single-variant martensite (S→S)

During a transformation, *u* and *ξ* are subjected to change and the resultant variables are given in Equations (2) and (3), as below:

(2) $$\dot{u}={\dot{u}}^{AS}+{\dot{u}}^{SA}+{\dot{u}}^{SS}$$

(3) $${\dot{\xi }}_{s}={\dot{\xi }}_{S}^{AS}+{\dot{\xi }}_{S}^{SA}$$

The reorientation occurs at constant *ξ*, therefore,$\;{\dot{\xi }}_{S}^{SS}=0$. Each of these transformations are assumed to occur in a region of the control-variable hyperplane *τ*-*T*, where *τ* is the Kirchhoff stress and *T* is the temperature.

#### 2.3.1. Conversion of Austenite into Single-Variant Martensite (A→S)

To model stress-induced (pressure-dependent) transformation, a Drucker–Prager-type loading function (Equation (4)) is introduced:

(4) $$F^{AS}\left(\tau ,T\right)=\|t\|+3\propto p-C^{AS}T$$

where t is the deviatoric stress; p is the pressure; $C^{AS}$ and ∝ are material parameters; ‖t‖ is the Euclidean norm of the term. The initial (Equation (5)) and final (Equation (6)) transformation functions are expressed as:

(5) $$F_{s}^{AS}=F^{AS}-R_{s}^{AS}$$

(6) $$F_{f}^{AS}=F^{AS}-R_{f}^{AS}$$

where,

(7) $$R_{s}^{AS}=\left[\sigma_{s}^{AS}\left(\sqrt{\frac{2}{3}}+\propto \right)-C^{AS}T_{s}^{AS}\right]$$

(8) $$R_{f}^{AS}=\left[\sigma_{f}^{AS}\left(\sqrt{\frac{2}{3}}+\propto \right)-C^{AS}T_{f}^{AS}\right]$$

where, $\sigma_{s}^{AS}$, $\sigma_{f}^{AS}$, $T_{s}^{AS}$, and $T_{f}^{AS}$ are material parameters. The condition for this transformation is assumed to be: $F_{s}^{AS}>0$; $F_{f}^{AS}<0$; ${\dot{F}}^{AS}>0$.

#### 2.3.2. Conversion of Single-Variant Martensite into Austenite (S→A)

Similar to the above transformation, a Drucker–Prager-type loading function (Equation (9)) is considered here also:

(9) $$F^{SA}\left(\tau ,T\right)=\|t\|+3\propto p-C^{SA}T$$

where $C^{SA}$ is also a material parameter. The initial (Equation (10)) and final (Equation (11)) transformation functions are expressed as:

(10) $$F_{s}^{SA}=F^{SA}-R_{s}^{SA}$$

(11) $$F_{f}^{SA}=F^{SA}-R_{f}^{SA}$$

where,

(12) $$R_{s}^{SA}=\left[\sigma_{s}^{SA}\left(\sqrt{\frac{2}{3}}+\propto \right)-C^{SA}T_{s}^{SA}\right]$$

(13) $$R_{f}^{SA}=\left[\sigma_{f}^{SA}\left(\sqrt{\frac{2}{3}}+\propto \right)-C^{SA}T_{f}^{SA}\right]$$

where, $\sigma_{s}^{SA}$, $\sigma_{f}^{SA}$, $T_{s}^{SA}$, and $T_{f}^{SA}$ are material parameters. The condition for this transformation is assumed to be: $F_{s}^{SA}<0$; $F_{f}^{SA}>0$; ${\dot{F}}^{SA}<0$. For a stress-free state, $T_{f}^{SA}$ is the temperature above which only the austenite phase is stable, while $T_{f}^{AS}$ is the temperature below which only the martensite is stable.

#### 2.3.3. Reorientation of the Single-Variant Martensite (S→S)

To model the reorientation process for non-proportional stress change (directional or rotational), the Drucker–Prager-type loading function (Equation (14)) is set as:

(14) $$F^{SS}=\|t\|+3\propto p-C^{SS}T$$

(15) $$F_{s}^{SS}=F^{SS}-R_{s}^{SS}$$

where,

(16) $$R_{s}^{SS}=\left[\sigma_{s}^{SS}\left(\sqrt{\frac{2}{3}}+\propto \right)-C^{SS}T_{s}^{SS}\right]$$

where, $\sigma_{s}^{SS}$—stress at reorientation of single-variant martensite,$\;C^{SS}$ and $T_{s}^{SS}$ are material parameters. The condition for the activation of reorientation is assumed to be: $F_{s}^{SS}>0$.

### 2.4. Numerical Simulations

Compared to most metallic materials, NiTi material is difficult to model because of the complex mechanical behavior as discussed earlier. In this study, FE modelling/simulation was performed using the package ANSYS Workbench 2019. The Auricchio model, which defines the shape memory material properties, was utilized coupling with custom material plasticity data implementation.

For an easier understanding of the phase transformations and stiffness variations, the superelasticity concept under pure mechanical effect and isothermal conditions (22 °C) was examined in this work. In other words, the stress-induced martensitic transformation (SIMT) was studied, ignoring all thermal effects involved. Initially, a mesh convergence study was implemented to obtain the right mesh size, resulting in good result convergence without consuming large computational resources. The model was then validated using a set of experimental data from Jiang and Li [95]. Data from an experimental study by Deurig et al. [96] were used to validate the coupled superelastic–plastic behavior of the model. The validated model was then used to study the effect of porosity in the superelastic region. In the last section, a microscale material model was used to study how the martensite volume fraction could affect the stiffness or Young’s modulus of the NiTi material and predictions were compared with the macroscale results.

Table 1 shows the three different material data sets that were used for the analyses. The density was assumed to be the same for the three materials as 6.45 g/cm^3^. For the macroscale study, a cube of geometry 5 × 5 × 5 mm was modelled with uniaxial mechanical loading/unloading (tension/compression) for a deformation of 0.5 mm (10% strain in fully dense model) at a constant strain rate of 0.1 min^−1^. The bottom of the cube was fully constrained (fixed).

**Figure 7:**
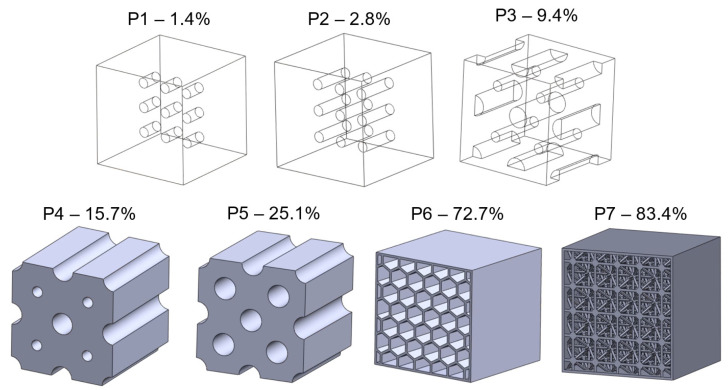
Porous structure designs showing the different types of cavities used; P1 to P5 varying degrees of cylindrical cavities; P6—enclosed honeycomb structure; and P7—lattice structure with 14-spokes per unit cell.

To study the effect of porosity, random cavities were incorporated in the 5 × 5 × 5 mm geometry, as shown in Figure 7, to represent different porosity levels (presented in Table 2). The porosity levels are calculated by taking the fraction of void volume to the cube volume. The two common types of porosities seen in L-PBF processed Nitinol are of process-induced and gas-induced types. Process-induced pores occur due to insufficient melting of powder particles; these are irregularly shaped and found around the edges. The gas-induced pores are usually spherical in shape and are found well inside the volume; they occur due to the trapping of ambient gases [13].

The microscale analysis was performed by creating a representative volume element (RVE) to simulate a NiTi microstructure. The microstructure was designed using a random particle RVE model in the Material Designer module in ANSYS Workbench 2019. The random particle RVE consists of spherical particles arranged randomly in a matrix material. Besides the position, the particle diameter was also randomly assigned following a uniform distribution between 20 μm to 70 μm (as in real NiTi microstructures). Due to the limitations imposed by the RVE periodicity and the need to create a suitable mesh, the particle diameter was forced to be larger than a program-controlled minimum diameter. It was, however, smaller than half the unit cell size. The RVE is assumed to have isotropic linear elastic matrix and particle properties. It is also assumed that the bonding between the particle and the matrix material is perfect.

**Figure 8:**
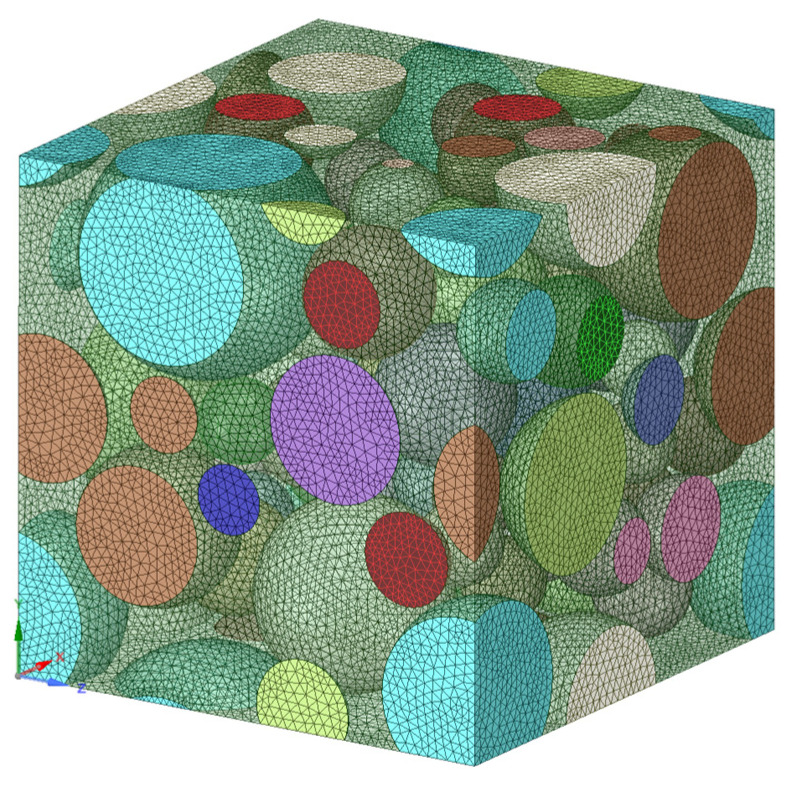
Random particle type RVE with a martensitic volume fraction of 0.9.

Since we needed to simulate a superelastic NiTi, the matrix was considered to be austenite at room temperature. As the material is stressed, the martensitic transformation is expected to reconfigure the microstructure into a fully detwinned martensite. This concept is simulated by gradually increasing the martensite (particle) volume fraction in the austenite matrix from 0.1 to 0.9 (0 being fully austenite; 1 being fully martensite microstructure). To ensure randomness in the position of particles and precise prediction of properties, five sample points were considered in the simulation for each MVF values. A sample meshed RVE containing 0.9 martensite volume fraction (MVF) is shown in Figure 8.

### 2.5. Mesh Convergence

**Figure 9:**
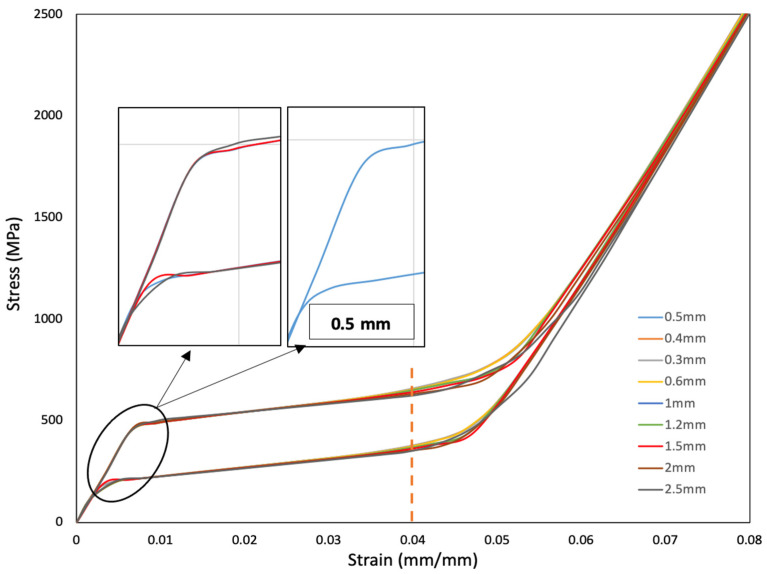
Stress–strain graph for mesh convergence study.

Mesh convergence is necessary to verify whether the generated mesh is capable of converging an appropriate solution. For this, material NT1 has been used. The superelasticity model considered traced almost similar curves for the different mesh sizes used (Figure 9). Therefore, to distinguish the effect of mesh sizes, two factors were considered based on theoretical observations—non-convergent regions and stress values at 4% strain.

Non-convergent regions—as seen in Figure 9, larger mesh sizes show some anomaly at the start/end of phase transformations. These can be considered as the non-convergent regions in the solution. It was observed that mesh sizes from 0.5 mm and below did not show any non-convergent region.

**Figure 10:**
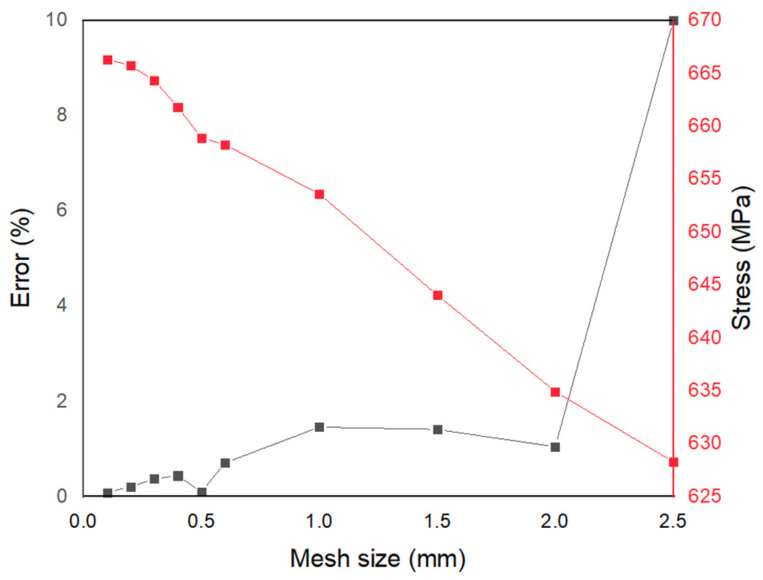
Stress values (red) at 4% strain and respective error % (black) for different mesh sizes.

Stress at 4% strain—in Figure 10, a noticeable difference in stresses can be seen around the strain value of 0.04 and above. Stress values at 0.04 strain for different mesh sizes are shown in Figure 10. It can be seen that the stress values stabilize with low error value around a mesh size of 0.6 mm. Even though a mesh size of 0.6 mm was efficient, 0.5 mm showed better convergence and lower error % (Figure 10). Lower mesh size gives better prediction; however, the computational time was almost doubled when the mesh size was reduced from 0.5 mm to 0.4 mm. Therefore, a mesh size of 0.5 mm was selected for macro-scale analysis. While it is not fully clear why the error is lower at 0.5 mm, this could be a discretization error from the mesh creation, numerical errors from integration, or rounding errors from numerical calculation.

## 3. Results

### 3.1. Model Validation

**Figure 11:**
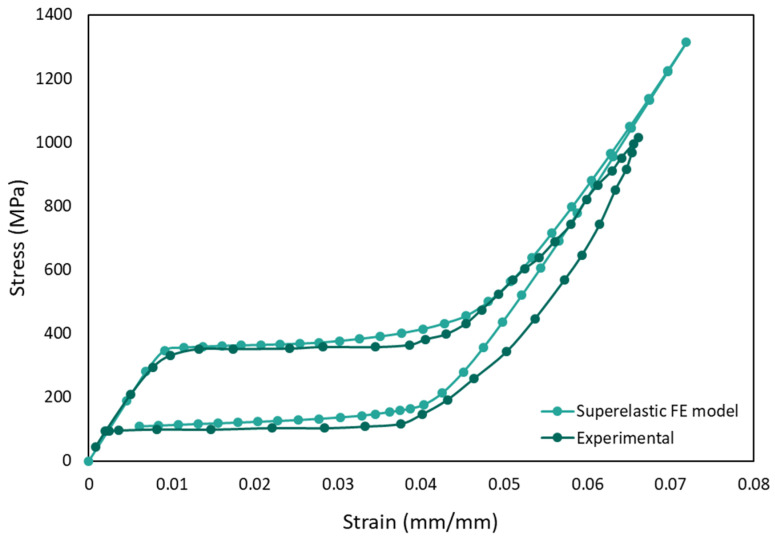
Validation of superelasticity model with actual experiment by Jiang and Li [95].

Initially, the Auricchio superelasticity model was validated using the experimental data from material NT2 following the same boundary conditions. As seen in Figure 11, the FEA model traces a similar curve to that of the experimental one.

Plasticity in superelastic NiTi is not of a prime interest in most applications, as the strain levels applied are generally well within the yield limit of the material. In the current work, a combined superelastic-plastic model was considered to simulate plasticity. The experimental data (NT1) from Deurig et al. [96] has been used for this validation. One of the limitations of the Auricchio model is that the plastic data cannot be coupled in the material data, as it is composed exclusively for superelastic behavior. Therefore, non-linear isotropic hardening data from the experiment was input into the material data to simulate the plastic region, and two discrete simulations were performed with different strain limits under the same straining conditions. After obtaining the plasticity and superelasticity data discretely, the output data were combined to represent the coupled mechanical behavior.

**Figure 12:**
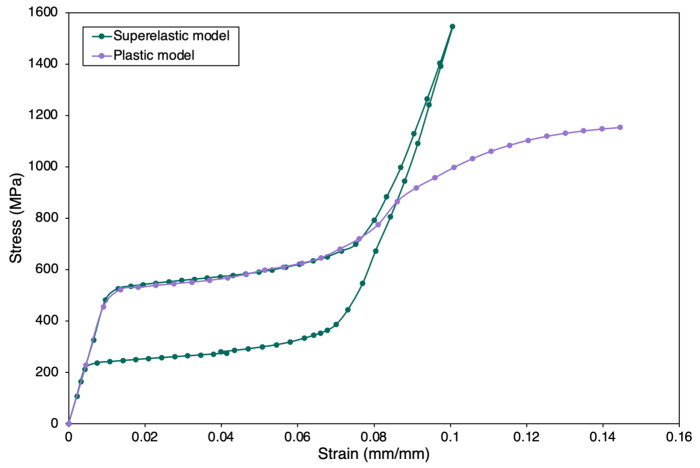
Discrete superelastic model and plastic model simulated results.

**Figure 13:**
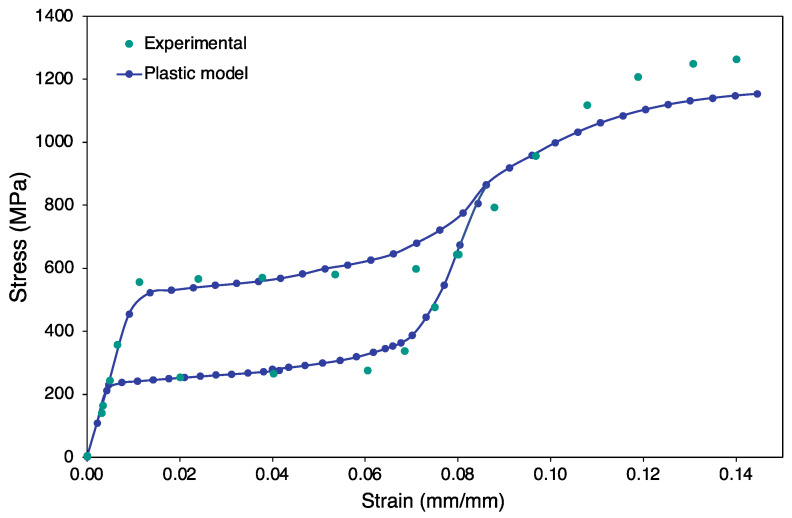
Comparison of experimental data and combined plastic model.

This approach of combining the output data was based on the concept that the initiation and evolution of two distinct yield profiles can be used to fully capture the martensitic transformation and plastic slip yield simultaneously [97,98,99]. A detailed model can include two limit functions to define both the phase transformation domain and plastic domain for predicting cycling effects and accumulation of inelastic strains. As presented in Figure 12, the plastic region always lies after a complete martensitic transformation, inferring that the superelastic phenomenon is distinct (analogous to the elastic region in steel) until the strain limit for plastic deformation has been reached. The plastic deformation was modelled to be triggered after the martensite phase was attained in the matrix (Figure 12). The coupled behavior of the FE model is shown in Figure 13; the curve traced is close in shape and magnitude to that of the experiment with slight stress offsets.

### 3.2. Response to Strain Levels

**Figure 14:**
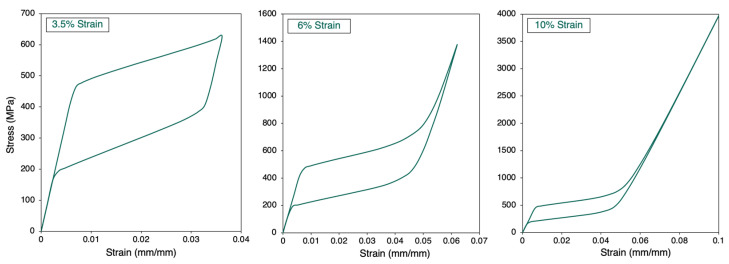
Stress–strain curves for different strain levels: 3.5%, 6%, and 10%.

The response of the model to different strain levels was explored under a constant strain rate of 0.1 min^−1^ using the material NT1. As seen in Figure 14, when the cube experienced a low strain of 3.5%, a partial phase transformation occurred, similar to what is found in an actual mechanical test. A 6% strain generated a complete martensitic transformation, while a 10% strain progressed with straining in the elastic region of detwinned martensite phase after completing the phase change.

### 3.3. Asymmetry in Tension and Compression

**Figure 15:**
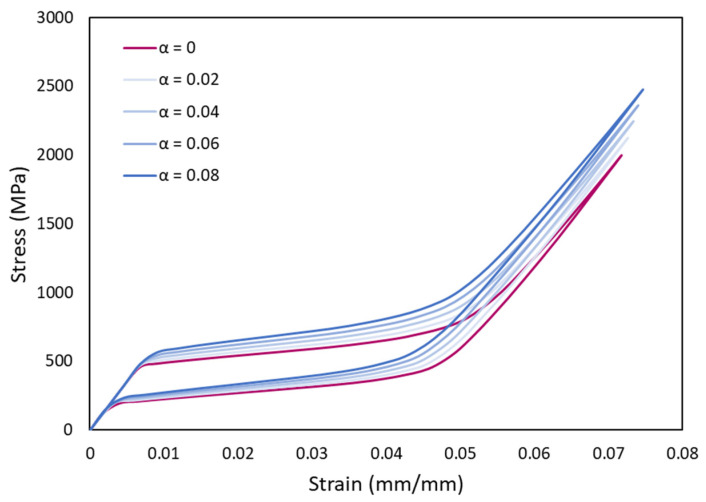
Stress–strain curves showing asymmetry between compression and tension.

It is often observed that the stress–strain curves generated during actual compression tests are slightly different when compared to the tension tests. This asymmetry can also be simulated in the Auricchio model using a factor, α as defined in Equation (1). The simulation was performed using material NT1. A is varied from 0 (symmetric) to 0.08, where α = 0 represents tension mode stresses. Figure 15 shows the asymmetric variation of compression curves with respect to tension, where the compression mode stresses are higher compared to the tension (α = 0).

### 3.4. Compression of Porous Structures

**Figure 16:**
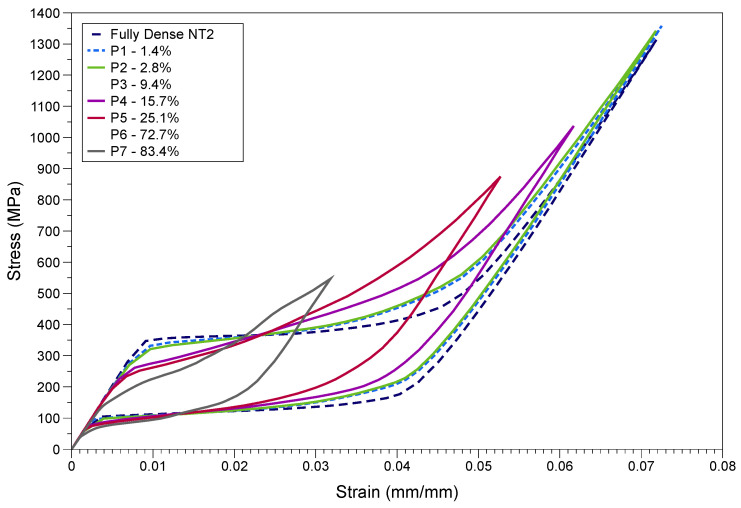
Simulated stress–strain curves for fully dense and porous NiTi structures.

The superelastic FE model was used to simulate the mechanical behavior of the porous NiTi structures shown in Figure 7, using material NT2. All input and boundary conditions were maintained to be similar to that of the fully dense part. The stiffness of the structure was calculated using the slope (martensitic elastic modulus) of the loading curve after complete phase transformation. The unloading curve is of less interest, as this is highly affected by the hysteresis that occurs. The generated stress–strain curves are shown in Figure 16.

**Figure 17:**
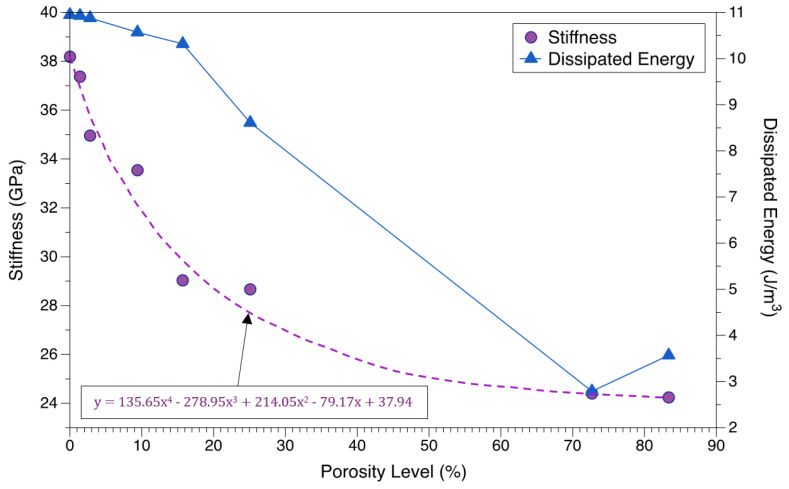
Variation of stiffness and energy dissipated per unit volume per cycle, with the increasing levels of porosity.

As shown in Figure 17, the structural stiffness was found to decrease drastically initially until 15% porosity, after which the gradient lowered and small reductions in stiffness were noted. The reduction in stiffness was found to be about 14 GPa for 83.4% porosity. Overall, the decrement in elastic modulus of the structure was seen to be more of an exponential trend. In contrast to the Gibson–Ashby analysis [100] of porous structures, which states a quadratic relation between the elastic modulus and porosity, the current study reveals a fourth order polynomial as shown in Figure 17. This could be due to some geometric effect (for instance, lattice vs cylindrical voids) in play when the material deforms. As the porosity levels increased to 83.4%, the dissipated energy ($W_{D}$) decreased by about 8 J/m^3^ accompanying a reduction of about 200 MPa in stress levels and a reduction of about 0.063 in strain levels. No particular trend could be interpreted between the levels of porosity and this reduction in dissipated energy. These factors play a major role in depicting the efficacy for heat pump applications.

**Figure 18:**
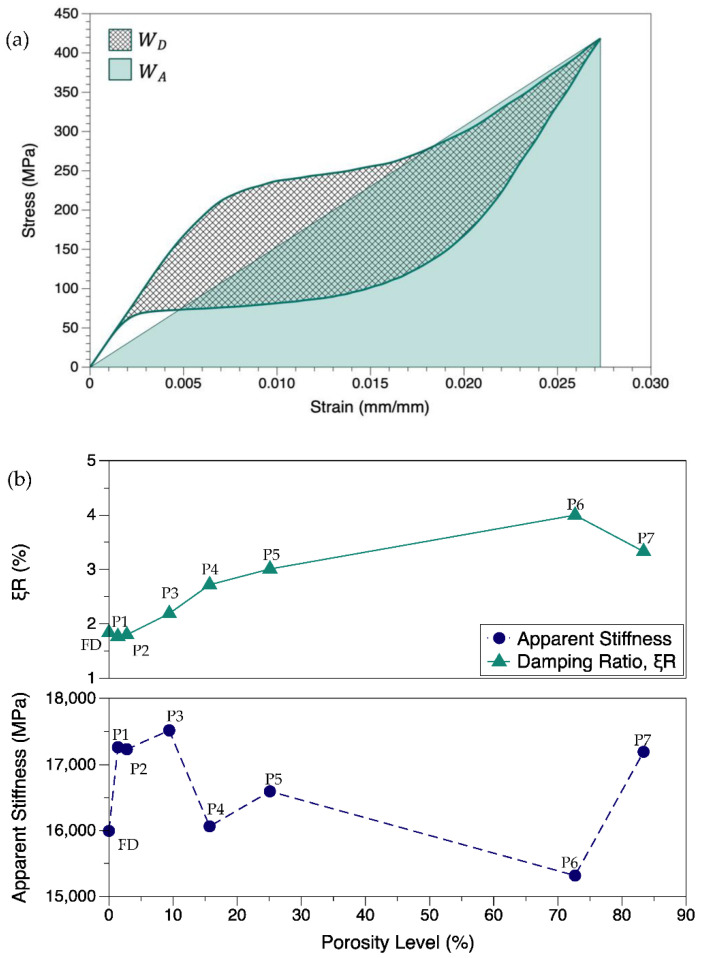
(**a**) Illustration of obtaining the dissipated and absorbed energy from stress–strain curves; (**b**) variation of damping ratio (ξR) and apparent stiffness with the increasing levels of porosity.

For the same porous structures, the energy absorbed ($W_{A}$) during a linear stress loading for the same maximum force and deformation was also obtained using the methodology shown in Figure 18a. $W_{A}$ and $W_{D}$ can be used to calculate the damping ratio (ξR), also known as loss factor as in Equation (17), to estimate the damping property of the structure when an external stress load is given. The slope of the $W_{A}$ triangle gives the apparent stiffness of the structure which considers the maximal strain point [101]. The damping ratio and apparent stiffness are of high relevance to shock absorption applications.

(17) $$\xi R=\frac{1}{4\pi }\frac{W_{D}}{W_{A}}$$

Referring to Figure 18b, the damping ratio was generally found to increase when porosity increased, and a sudden fall was noted for 83.4% porosity. There was also a minor decrease in ξR for low porosities compared to the fully dense structure. Although no specific trend was found between the apparent stiffness and porosity levels, there was a general rise in stiffness values for structures with more uniformly spread cavities.

### 3.5. MVF vs. Elastic Modulus

In this section, the effect of martensitic phase transformation in the matrix on material stiffness was explored using a microscale RVE model. The mechanical properties of NiTi are primarily dependent on the material composition and phase structures, resulting from various factors including processing techniques and thermal and mechanical histories. The current study considers the material composition exclusively to estimate an upper bound and a lower bound for the material stiffness of NiTi alloys. As presented in the ASM handbook [53], the composition of Ni varies from 54 to 56 wt.% for NiTi alloys, and therefore these composition limits were taken into account. The Young’s modulus for Ti is lower (110 GPa) compared to Ni (210 GPa), hence higher Ni content in NiTi would result in higher elastic moduli. For 56 wt.% NiTi, the stiffness data from ASM handbook [53] were used to represent the upper bound. However, for the lower bound, data from NT3 were used as the material presents a lower $E_{M}$ and there were no sufficient data for the 54 wt.% NiTi in ASM handbook. Presenting the data using these upper and lower bounds (Table 3) will enhance the understanding of how the stiffness may vary within the compositional range and martensite phase volume fraction.

**Figure 19:**
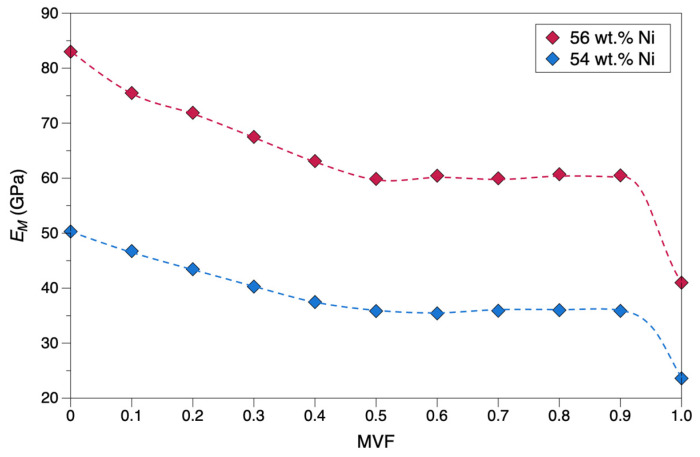
Effect of martensite phase evolution on the stiffness of NiTi for the two Ni contents.

The modulus of elasticity (stiffness) is calculated on the premise that the RVE presents a homogenized microstructure. Initially the matrix is fully austenite (0 MVF), and the resultant stiffness is equal to $E_{A}$. As martensitic transformation takes place, the martensite phase starts to form in the microstructure, gradually decreasing the stiffness as shown in Figure 19. When 1 MVF is reached, the material is fully martensite, and the resultant stiffness is equal to $E_{M}$. Irrespective of the material composition, a gradual decrease of stiffness was observed until 0.5 MVF, and then the stiffness starts to stabilize until 0.9 MVF, and then decreases rapidly towards 1 MVF point.

## 4. Discussion

In the actual experiment, the hysteresis between loading and unloading curves is more than the simulated results. This can be due to the reorientation of martensite phase (explained in Section 1.1. on Kinematics of phase transformation) and the presence of residual martensite phase while unloading. In the current FE model, the possibility of residual martensite is ignored, and only the reorientation of martensitic phase is considered, resulting in a lower hysteresis. The hysteresis and stress levels in actual experiments will gradually decline and stabilize after a certain number of cycles [97]. The validated model was then used to study the mechanical behavior in more detail.

### 4.1. Mechanical Strain

When the compression was simulated (Figure 14) for different strain levels, partial and complete transformations were observed. Partial transformation and recovery are also associated with martensite reorientation, as that in complete transformation (within the martensitic elastic limit). Since the plasticity is ignored in this discrete model, the effect of plastic deformation is not accounted for.

When tension and compression modes for the same strain conditions are observed (Figure 15), we can see higher stress values in compression compared to the tension mode. Generally, a volume rise is associated with the martensitic reorientation and transformation, that results in a crystallographic asymmetry [36,102]. This induces a lower stress requirement to generate the transformation in tension than in compression mode. The α-value depends on the material properties, specifically the thermal and mechanical history that could affect the lattice.

### 4.2. Structural Porosity

Contrary to the 14 GPa drop in stiffness observed when porosity was increased to 83.4%, in bone-implant applications, the reduction in stiffness is much higher for similar levels of porosities. This can be attributed to two reasons. One reason is the fact that the current model does not include the martensite elastic modulus as an input parameter, rather the elasticity of martensite depends on austenite stiffness, elongation strain, and transformation parameters. The second reason is suspected to be the cavities/pore designs considered in the current study. In actual components, these are more lattice-like and uniformly spread throughout the volume, in contrast to the ones considered in the current study.

In renewable energy application for heat pumps, these porosity levels increase the surface area of the structure that contacts the working fluid, resulting in increased heat conduction/transfers. In conventional designs, the NiTi component is designed with channels inside to facilitate the passage of working fluid. These channels are less efficient compared to the higher porosities that can be engineered via L-PBF. The performance of a heat pump is generally represented by the coefficient of performance (*COP*) in Equation (18). The work done by the material (*Q*_*out*_) can be determined by calculating the area contained within the martensitic transformation region (enclosed by the loading and unloading curve) on the stress–strain curve. This is also referred to as the dissipated energy per cycle or enthalpy [103]. *Q*_*out*_ is the latent heat energy required for the martensitic transformation (obtained from Differential Scanning Calorimetry analysis).

(18) $$COP=\frac{Q_{out}}{Q_{in}}$$

It was seen that the porosity levels induced lower enclosed area under the martensitic transformation curve (indicating lower *Q*_*out*_). As mentioned earlier, the simulations of these porous structures were conducted for 0.5 mm deformation, similar to that of the fully dense component. Structural porosity results in a lower resultant cross-sectional area and length compared to the fully dense part, and this has contributed towards an effective reduction in the stress and strain levels. No significant trend was noted for energy dissipation with respect to porosity. This could be attributed to the possibility of different resultant cross-sectional areas and lengths for the same levels of porosity. It should also be noted that a further reduction in mechanical properties takes place when the component is deployed in the high temperature ambient conditions prevalent in heat pumps. For example, a decline of about 10 GPa in ultimate strength was observed when temperature was increased by 200 K from room temperature [104].

The dissipated energy is not a material property as it hugely depends on strain levels, and the associated microscale mechanisms are generally non-linear. When deformation progresses, the strain increases, requiring more energy which, at the end, is dissipated. In a practical aspect, this maximum strain should be decided appropriately to get higher *COP*. A complex porous structure, which can be realized via L-PBF, is highly beneficial in reducing material volume compared to a fully dense component, however, the lower cross-sectional area affects the *Q_out_* and in turn affects the *COP*. However, a complex structure such as P6 and P7 might have lower stress limits that could affect the structural strength and operational integrity. Compared to most lattice structure topologies, the design of P7 (14-spoke lattice) possesses a higher mechanical strength (Poisson’s ratio and elastic modulus) [105]. To summarize, an all-embracing balance should be devised between the surface area (porosity) in contact, stress compensations, and maximum strain levels in order to achieve a high *COP*.

The dissipated energy due to mechanical hysteresis is also highly relevant in the shock absorption applications such as vibration, impact, and seismic shock dampers. These applications often undergo dynamic loading. The mechanical hysteresis of NiTi enables absorption of the external load. As shown in Equation (17), ξR gives an estimate of the damping capabilities of the NiTi structure. The damping is also enabled by martensitic reorientations and is found to depend on the size and structural design [102,106].

The loss factor ξR is usually found to vary between 8% and 53% for full-scale analysis. In the current study, due to the small sample size, ξR is much lower (1.8% to 4.0%). It can be seen that the porous structures provide better damping property. Besides SIMT, martensitic reorientations and twin boundary motions also mechanize the damping/self-centering effects [102,107]. The two latter mechanisms are not considered in this study. The porous structures are susceptible to anisotropic mechanical responses such as localized plastic deformation or de-twinning effects. These can also affect the hysteresis region, which can affect both $W_{A}$ and $W_{D}$. A higher $W_{A}$ and lower $W_{D}$ results in higher ξR. Proper selection of heat treatment parameters can enable lower phase transformation stresses and austenitic elastic moduli. This can enable a lower $W_{D}$.

For porous structures, the out-of-plane elastic modulus will be different to the in-plane elastic modulus [107]. Similarly, these could be different from the martensite elastic modulus. Therefore, the apparent stiffness that takes into account the maximum force and deformation represents the effective stiffness of the structure during operation. The apparent stiffness will generally increase, when the material undergoes cyclic loading eventually leading to mechanical stability. This factor helps in identifying the mechanical strength of the structure for shock-related applications. In the current study, the similar deformation input resulted in varying degrees of strains and stresses for different porosity levels. This has resulted in different apparent stiffness values ranging 15.3 GPa to 17.5 GPa. In general, the values were comparatively higher than that of the fully dense part.

Due to localized deformation heterogeneity in porous structures, the geometry of the component is critical for apparent stiffness and loss factor calculations. This might have played a key role in generating a lower apparent stiffness for P6 and a lower ξR for P7. To summarize, the structures with 10% to 30% porosities showed a good balance between structural stiffness, $W_{D}$, ξR, and apparent stiffness.

### 4.3. Martensite Evolution vs. Stiffness

**Figure 20:**
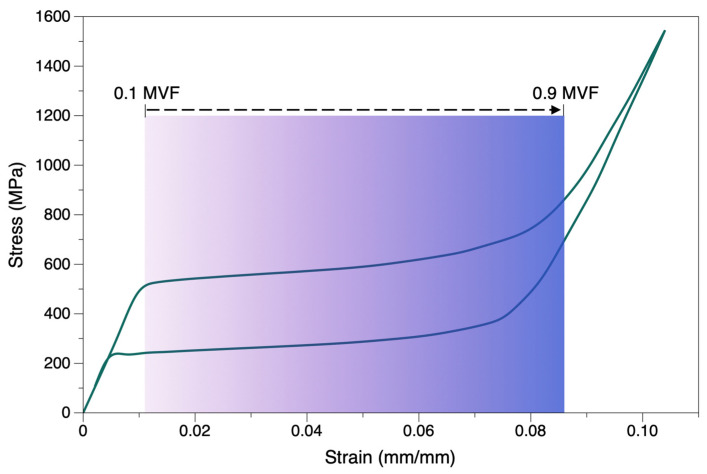
Stress–strain curve to illustrate the evolution of martensite phase during stress-induced transformation (SIMT) via stiffness slopes.

An RVE-based analysis of elastic modulus variation with respect to martensite evolution in the microstructure was investigated (Figure 19). A gradual reduction until 0.5 MVF and then a level off was seen until 0.9 MVF. This might be because the particle-matrix system is homogenized, and a value of 0.5 MVF denotes martensite phases being uniformly saturated until 0.9 MVF (almost martensite). Referring to the stress–strain curves in Figure 20, it can be seen when phase transformation occurs. The crystal structure evolves from BCC to monoclinic structure, causing an increase in strain with a marginal rise in stress. We can see that near the end of martensitic transformation (0.9 < MVF < 1), a larger change in stress occurred compared to initiation (0.1 MVF) or propagation (0.2 to 0.8 MVF) of martensitic transformation. Until the matrix is almost entirely martensite, the austenite stiffness is predominant. This dominance is rapidly degraded as the matrix transforms to complete martensite (0.9 < MVF < 1), and this is noted by a rapid decrease in stiffness, attaining a value equivalent to the Young’s modulus of martensite phase.

The initial transformation in superelastic NiTi is partially triggered by the surface energy to create an interface, and partially via the elastic energy of the accommodation of martensite in the austenite matrix. This causes a reduction in free energy, and therefore, the nucleation of martensite phase usually requires a higher driving force than the subsequent propagation of the austenite-martensite interface. The size and shape of a martensite nucleus is governed by this free energy reduction [60,63]. This could also explain the rapid change in modulus values (0.7 to 0.9 MVF) noted in the stress–strain curves. It is noteworthy that the martensite evolution is high in the regions of stress concentrations in porous structures. Under stresses, these regions will have a higher MVF compared to other regions in the bulk. This principle is also applicable in the case of porous structures, where martensite evolution will be abrupt around the pore regions.

## 5. Conclusions

An Auricchio material model was used to simulate the mechanical behavior in NiTi alloys and perform macroscale study of the mechanical response at various porosity levels. The model was shown to provide close predictions of real material behavior, capable of responding to different strain levels, and asymmetry in tension and compression. It was found that an increase in porosity of up to 83.4% resulted in a reduction of about 14 GPa in the structural stiffness. The dissipated energy during the phase transformation was reduced by about 8 J/m^3^, along with a stress reduction of about 200 MPa and strain decrease of 0.063. The damping ratio increased from 1.8% to 4.0% with an increase in porosity. The apparent stiffness for damping operations showed no trend, however, exhibited a general rise in porous samples compared to the fully dense sample.

The stiffness for the austenite phase is generally higher than the martensite phase. The SIMT results in temporary softening of the material. A microscale model using RVE was created to study the gradual decline in stiffness as the martensite phase evolved (increasing the MVF). This was then compared to investigate the response in macroscale analysis; the larger curvature in stress–strain curve near the end of transformation corresponded to a sudden drop in Young’s modulus. It is known that L-PBF process parameters affects the microstructure and chemical composition of NiTi. This affects the transformation characteristics, which results in varied energy absorption levels in the structure. This will also affect the operational limits in heat pump applications.

The current model was not built to simulate the thermal or stabilization effect during mechanical cycling. Usually, during the first cycle, the microstructure is modified and self-oriented, and a residual deformation is observed. This residual deformation will propagate until the mechanical curve stabilizes. For shock absorption applications, a lower residual deformation and higher strain recovery is desired. The model is not sufficiently sensitive to the martensite stiffness changes. This low sensitivity was reflected in a lower reduction of structural stiffness for the same porosity levels compared to real-world observations. The developed model does determine how the phase structure results in specific mechanical responses, which can be run in a reasonable period. Thus, this modelling method is highly useful for determining the stress–strain response of NiTi SMAs. Future work directions could include the development of a combined multi-physics-based model that accounts for stabilization effect, thermal effect, MVF and local heterogeneity. Another area to explore is the localized SIMT for different porous designs, preferably via full-field strain analysis. These data can be used to validate developed models, including the proposed mentioned multi-physical model. The results from the work in this paper and these future developments provide a needed, more thorough insight into the application-based research of NiTi.

## Figures and Tables

**Table 1:** Material data [11,95,96] used in the FEA models for determining the stress–strain responses.

Material	$E_{A}\;(\mathrm{GPa})$	ν	$\sigma_{s}^{AS}\;(\mathrm{MPa})$	$\sigma_{f}^{AS}\;(\mathrm{MPa})$	$\sigma_{s}^{SA}\;(\mathrm{MPa})$	$\sigma_{f}^{SA}\;(\mathrm{MPa})$	$\varepsilon_{L}$
NT1	71.1	0.3	500	700	400	200	0.044
NT2	41	0.33	380	390	145	110	0.040
NT3	50.3	0.3	556	643	315	246	0.075

**Table 2:** Levels of porosity considered in the study; a fully dense cube volume is 125 mm^3^.

Sample	Porosity (%)	Void Volume (mm^3^)
Fully Dense (FD)	0	0
P1	1.4	1.77
P2	2.8	3.53
P3	9.4	11.78
P4	15.7	19.63
P5	25.1	31.42
P6	72.7	90.85
P7	83.4	104.21

**Table 3:** Upper/lower bound values of stiffness with respect to Ni content in NiTi used in RVEs.

	$E_{A}\;(\mathrm{GPa})$	$E_{M}\;(\mathrm{GPa})$	ν
Upper bound (56 wt.% Ni)	83	41	0.3
Lower bound (54 wt.% Ni)	50.30	23.59	0.3

## Data Availability

Not applicable.

## Appendix A

### Elastic Modulus Estimation from Martensite Volume Fraction

The effective elastic modulus of the material is conventionally calculated as below:

(A1) $$\mathrm{Voigt}\;\mathrm{approach}:\;E_{eff}=V_{m}E_{M}+\left(1-V_{m}\right)E_{A}$$

(A2) $$\mathrm{Reuss}\;\mathrm{average}\;\mathrm{approach}:\;E_{eff}={\left\{\frac{V_{m}}{E_{M}}+\frac{\left(1-V_{m}\right)}{E_{A}}\right\}}^{-1}$$

where, $V_{m}$ is the volume fraction of martensite and $E_{eff}$ is the effective Young’s modulus. A phase transformation flow rule (Equation (A2)) was proposed by Auricchio et al. [31] involving the martensite volume fraction, f(x,t):

(A3) $$\dot{f}\left(x,t\right)=\frac{\partial f}{\partial t}=V_{t}\left(f_{t}\left(x,t\right)-f\left(x,t\right)\right)$$

where, $f_{t}\left(x,t\right)$ is the driving force for phase transformation and $V_{t}$ is the maximum transformation rate. This first order differential equation (Equation (A2)) includes a delay between the driving force and the evolution of martensitic ratio. This simple flow rule considers only the two phases and therefore, the complexity of real phase transformation is not fully reproduced.
